# Supplementary material for: Machine learning identifies risk factors associated with long-term sick leave following COVID-19 in Danish population
Source: Commun Med (Lond). 2023 Dec 20;3:188. doi: 10.1038/s43856-023-00423-5 (PMC10733276; doi:10.1038/s43856-023-00423-5)
Supplement: Supplementary file 1 — Supplementary Information [file 43856_2023_423_MOESM1_ESM.pdf]

Machine learning identifies risk factors associated with long-term sick leave following COVID-19 in Danish population – Supplementary Information

Kim Daniel Jakobsen[1], Elisabeth O'Regan[1], Ingrid Bech Svalgaard[1], Anders Hviid[1,2]

1: Department of Epidemiology Research, Statens Serum Institut, Copenhagen, Denmark

2: Pharmacovigilance Research Centre, Department of Drug Design and Pharmacology, University of Copenhagen, Copenhagen, Denmark

Correspondence to:
Kim Daniel Jakobsen, Department of Epidemiology Research, Statens Serum Institut, Artillerivej 5, DK-2300 Copenhagen, Denmark / kija@ssi.dk / phone: +45 32 683 659

Table of Contents

Supplementary methods 1: Causal forest technical details 3

Supplementary methods 2: Sensitivity analyses 5

Supplementary results 1: Analysis of more three-way interactions........ 6

Supplementary results 2: Sensitivity analyses 6

Supplementary discussion........ 7

Supplementary table 1: Characteristics of 88,818 study participants who obtained a positive or negative RT-PCR test for SARS-CoV-2. 9

Supplementary table 2: Subpopulation counts for combinations of age, high BMI, and depression.... 11

Supplementary table 3: Subpopulation counts for combinations of sex, depression, and high BMI 12

Supplementary table 4: Covariate balance between study participants with and without a positive RT-PCR test........ 13

Supplementary figure 1: Causal tree example........ 14

Supplementary figure 2: Causal forest variable importance measure 15

Supplementary figure 3: Estimated risk differences for combinations of sex, high BMI, and age........ 16

Supplementary figure 4: Estimated risk differences for combinations of sex, age, and depression...... 17

Supplementary figure 5: Estimated risk differences for combinations of sex, age, and health conditions........ 18

Supplementary figure 6: Distribution of estimated propensity scores 19

Supplementary figure 7: Covariate balance – Love plot 20

Supplementary figure 8: Covariate balance – eCDF plots 21

Supplementary figure 9: Covariate balance – eCDF plots 22

Supplementary figure 10: Covariate balance – eCDF plots 23

Supplementary figure 11: RT-PCR test sensitivity analysis - variable importance........ 24

Supplementary figure 12: Hyperparameter sensitivity analysis - variable importance 25

Supplementary References 26

Supplementary methods 1: Causal forest technical details

Let Y be the observed outcome, W the binary treatment indicator, $Y^{(w)}$ the potential outcome under treatment w, and X the covariates. The causal forest (CF) method seeks to estimate the conditional average treatment effect (CATE) at $X = x$,

$$\tau(x) = E\left[Y^{(1)} - Y^{(0)} \mid X = x \right].$$

The identification of CATE's requires standard causal inference assumptions to hold. Specifically, we make the following assumptions:

1. $0 < P(W = 1 \mid X = x) < 1$ (Positivity)
2. $Y = Y^{(W)}$ (Consistency)
3. $Y^{(0)}, Y^{(1)} \perp\!\!\!\perp W \mid X$ (Conditional exchangeability)

CF utilises a metalearner to estimate treatment heterogeneity. These are meta-algorithms that leverage prediction models to estimate conditional causal effects. Specifically, it uses the R-learner [1]. The R-learner is based on the following relationship between the outcome and treatment indicator,

$$Y - m(X) = \big(W - e(X)\big)\tau(X) + \varepsilon$$

where ε is a mean zero error term and

$$m(x) = E[Y \mid X = x], e(x) = E[W \mid X = x].$$

The CATE can thus be characterised through the following loss-based representation,

$$\tau(x) = argmin_{\tau} E\left[\Big(\big(Y_i - m(X_i)\big) - \big(W_i - e(X_i)\big)\tau \Big)^2 \,\Big|\, X_i = x \right].$$

CF estimates CATEs by minimising the weighted empirical least squares loss with weight function $\alpha_i(x)$,

$$\hat{\tau}(x) = argmin_{\tau} \sum_{i=1}^{n} \alpha_i(x) \left(\left(\left(Y_i - \hat{m}^{(-i)}(X_i)\right) - \left(W_i - \hat{e}^{(-i)}(X_i)\right)\tau \right)^2 \right).$$

This minimisation problem contains three unknown quantities, $\alpha_i(x)$, $m(X_i)$, and $e(X_i)$. As such, we need to predict these quantities for each observation i. Prediction happens in two steps. In the first step, regression forests are used to predict the conditional outcome and propensity score for each observation. $\hat{m}^{(-i)}(X_i)$ is the predicted outcome at the covariates of the i-th sample, obtained from

the regression forest using only trees not containing the i-th sample (out-of-bag). Similarly, $\hat{e}^{(-i)}(X_i)$ is the out-of-bag predicted propensity to treat at the covariates of the i-th sample. In the second step, the weight function is predicted for each observation. The prediction algorithm is a modified version of random forest [2], using the splitting criterion from the generalised random forest algorithm [3]. Honest trees are grown by randomly sampling two disjoint subsamples, S and T, without replacement. The first subsample, S, is used to recursively partition the covariate space. Splits are axis aligned, and chosen greedily to maximise the difference between the CATE in each subset. Once the tree is grown, the second subsample, T, is used to populate the leaves of the tree. Assuming $L_b(x)$ is the set of samples from T falling in the same leaf as x within the b'th tree, the estimated weight function is given by

$$\alpha_i(x) = \frac{1}{B}\sum_{i=1}^{B} \frac{1\big(X_i \in L_b(x)\big)}{|L_b(x)|}.$$

The weight function adjusts the observed sample to look like a sample with $X_i = x$. Once we have obtained predictions of $\alpha_i(x)$, $m(X_i)$, and $e(X_i)$, CATE's are estimated by solving the loss-based minimisation problem,

$$\hat{\tau}(x) = \frac{\sum_{i=1}^{n} \alpha_i(x)\left(Y_i - \hat{m}^{(-i)}(X_i)\right)\left(W_i - \hat{e}^{(-i)}(X_i)\right)}{\sum_{i=1}^{n} \alpha_i(x)\left(W_i - \hat{e}^{(-i)}(X_i)\right)^2}$$

Given a CF model, treatment effect heterogeneity can be evaluated using Rank-Weighted Average Treatment Effect (RATE).[4] RATE takes a treatment prioritisation rule $S(X_i)$ and assesses the rules ability to prioritise individuals with the largest treatment effect. For any threshold $0 < u \leq 1$, define the targeting operator characteristic,

$$TOC(u; S) = E\big[\,Y_i(1) - Y_i(0) \mid F_S\big(S(X_i)\big) \geq 1 - u\,\big] - E[Y_i(1) - Y_i(0)].$$

RATE metrics are integrals over a weighted TOC curve,

$$\theta_\alpha(S) = \int_0^1 \alpha(u) TOC(u; S) du.$$

We used a RATE with $\alpha(u) = 1$, which corresponds to the area under the TOC curve (AUTOC). The RATE is estimated using terms from the AIPW estimator of the ATE. These AIPW scores, $\hat{\Gamma}_i$, can be expressed in terms of $\hat{\tau}^{(-i)}(X_i)$, $\hat{m}^{(-i)}(X_i)$, and $\hat{e}^{(-i)}(X_i)$,

$$\hat{\Gamma}_i = \hat{\tau}^{(-i)}(X_i) + \frac{W_i - \hat{e}^{(-i)}(X_i)}{\hat{e}^{(-i)}(X_i)\left(1 - \hat{e}^{(-i)}(X_i)\right)}\left(Y_i - \hat{m}^{(-i)}(X_i) - \left(W_i - \hat{e}^{(-i)}(X_i)\right)\hat{\tau}^{(-i)}(X_i)\right).$$

If there are no ties in $S(\cdot)$, the TOC estimator is

$$\widehat{TOC}(u; S) = \frac{1}{\lfloor un \rfloor}\sum_{j=1}^{\lfloor un \rfloor} \hat{\Gamma}_{i(j)} - \frac{1}{\mathrm{n}}\sum_{i=1}^{n} \hat{\Gamma}_i,$$

and the estimator for a RATE with weights $\alpha(u)$ is

$$\hat{\theta}_{\alpha}(S) = \frac{1}{\mathrm{n}}\sum_{i=1}^{n} \alpha\left(\frac{j}{n}\right)\widehat{TOC}\left(\frac{j}{n}; S\right).$$

If there are ties in $S(\cdot)$, the AIPW scores are averaged over tied observations. The RATE metrics provide tests for the hypothesis of treatment effect heterogeneity along the prioritisation rule S, using the null hypothesis $H_0\colon \theta_{\alpha}(S) = 0$. The AUTOC estimator $\hat{\theta}$ is asymptotically linear, so an asymptotically valid test for H_0 can be constructed using the bootstrap. With $\hat{\sigma}$ denoting the standard error of the bootstrap estimates and Φ the cumulative distribution function of the standard normal distribution, the P-value for H_0 is

$$2\Phi\left(-\frac{|\hat{\theta}|}{\hat{\sigma}}\right).$$

Supplementary methods 2: Sensitivity analyses

We conducted two sensitivity analyses. First, we evaluated the impact of false RT-PCR test results by rerunning our analysis 20 times using a modified exposure. Binny et.al. found RT-PCR sensitivity stays above 88% in the first two weeks post-infection, with a peak of 92.7%.[5] Skittrall et.al. report high specificity above 99%.[6] To evaluate the impact of false test results, we assumed a fixed sensitivity of 90% and a fixed specificity of 99%. Using these values, we solved for the number of false negatives (FN = 4112) and false positives (FP = 477) in the study population. These labels were assigned at random and the modified exposure had TP + FN in the exposed group, and TN + FP in the unexposed group. Second, we evaluated the impact of our choice of hyperparameters in the causal forest algorithm. We reran our analysis with all combinations of the following hyperparameters: sample fraction (0.3, 0.4, 0.5), mtry (10, 15, 20), minimum node size (5, 10, 20), honesty fraction (0.4, 0.5, 0.6). The algorithm has two hyperparameters (imbalance penalty and alpha) meant to limit

the amount of imbalance between the two child leaves from a split. Due to the low prevalence of several health conditions, we manually set these parameters to allow splitting along all covariates. These choices were kept for the sensitivity analysis.

Supplementary results 1: Analysis of more three-way interactions

We looked at more three-way interactions with age, sex, and additional health conditions, including chronic asthma, chronic or frequent headaches/migraines, PTSD, and diabetes (Supplementary figure 5). The interactions between age, sex, and headaches, PTSD, or diabetes showed significant uncertainty in the RD estimates for persons with the health condition. The interaction between age, sex, and chronic asthma showed an unexpected pattern between females with chronic asthma and age. The RDs increase sharply between age 20-40 years, with the highest AIPW RD observed for females with chronic asthma between 36-45 years (RD 10.4%, 95% CI 6.5% to 14.4%). The RDs drop for females with chronic asthma between 46-55 years (RD 6.4%, 95% CI 3.2% to 9.6%) and between 53-64 years (RD 8.9%, 95% CI 5.6% to 12.2%). In contrast, males with chronic asthma had zero risk difference up to an age of 45 years, with an increase for males with chronic asthma between 46-55 years (RD 5.5%, 95% CI 2.5% to 8.5%) and between 56-65 years (RD 4.2%, 95% CI 0.7% to 7.7%). RDs for males with chronic asthma were significantly higher than for males without chronic asthma in the 46-55 years age group (P-value 0.045). For females, those with chronic asthma have significantly higher RDs for age 36-45 years (P-value 0.002) and 56-65 years (P-value 0.045).

Supplementary results 2: Sensitivity analyses

Evaluating the impact of false RT-PCR test results, calibration measures showed similar results to the main analysis. The coefficient of the mean forest prediction was on average 1.00 (min 1.00, max 1.00) and the coefficient of the differential forest prediction was on average 0.61 (min 0.58, max 0.65). The variable importance measure consistently ranked the same five risk factors highest as in the main analysis. Age and high BMI were always ranked 1st and 2nd, however, education would sometimes rank higher than sex or depression, see Supplementary figure 11. The RD in the full population was slightly lower than in the main analysis (RD average 2.9, min 2.8, max 3.0). A similar pattern was observed for RD estimates along single risk factors, see Table 4. Accordingly, the RATEs are smaller than in the main analysis. For some modified exposures, fewer risk factors showed strong evidence for variability in RDs, specifically COPD or other lung disease, post-traumatic stress disorder, and chronic or frequent headaches had observed P-values above 0.05. Still, evidence for

heterogeneity in the total study population was strong, also along the risk factors identified as important by the variable importance measure. The RD estimates for the interaction between age, high BMI, and depression are shown in Figure 5. They show an increase with each covariate almost identical to the main analysis. Again, the RDs tend to be slightly lower compared to the main analysis. In summary, false RT-PCR test results due to imperfect sensitivity and specificity may have caused a small overestimation of the increased risk of full-time sick leave after COVID-19 infection.

Evaluating the impact of our choice of hyperparameters in the causal forest algorithm, the coefficient of the mean forest prediction indicated that the mean forest prediction was well calibrated for all combinations of hyperparameters (average 1.00, min 0.99, max 1.01). The coefficient of the differential forest prediction showed more variation (average 0.83, min 0.49, max 1.13), indicating that conclusions about the calibration of the heterogeneity estimates depend significantly on the choice of hyperparameters. Results on variable importance are similar to the RT-PCR sensitivity analysis. The five risk factors ranked most important are consistent across choices of hyperparameters. Of the four covariates ranked most important in the main analysis, sex ranks below education in 70 of 81 cases, while depression ranks below education in 36 of 81 cases see Supplementary figure 12. The RD estimate in the full population and along single risk factors changed only minimally across choices of hyperparameters, see Table 5. The same was the case for the interaction between age, high BMI, and depression, see Figure 5. Overall, there was no evidence that the estimated RDs were sensitive to the choice of hyperparameters in the causal forest algorithm, excluding parameters controlling the imbalance between child leaves in a split, which were kept fixed.

Supplementary discussion

For the age, sex, and chronic asthma interaction, the increase in RD varied by age, suggesting a high RD at a middle age between 36-45 years, with lower risk between both 26-35 years (4.1%) and 46-55 years (6.4%). Asthma has been reported as a risk factor for PCC in existing literature. Tsampasian et al. reported a significantly higher risk of developing PCC, with odds ratio 1.24 (95% CI 1.15 to 1.35),[7] while Jacob et al. reported an increased prevalence of long-term COVID-19 sick leave for persons with asthma (8.8%).[8] However, to our knowledge, previous literature has not reported on the interaction between age, sex, and chronic asthma.

In previous work by colleagues using the same cohort, age was treated as a categorical variable.[9] In the present work, age was kept as a continuous variable. This was chosen in part due to the goal of

estimating individual level causal effects, in part because the CF method treats all covariates as continuous. To work with categorical covariates, they must be manually one-hot encoded (or encoded using some other appropriate scheme).

Comorbidities were only registered if they were present before the RT-PCR test, as they might otherwise act as mediators of the COVID-19 effect on sick leave.

We note a few additional limitations to those discussed in the main discussion. First, with the variable importance measure, only one split per level is possible on categorical covariates. This limits the number of splits per tree. This risks a lower importance score for categorical covariates with few levels, compared with a continuous covariate with similar overall treatment effect. Particularly, age and education level will have more opportunities to be split along in each tree compared with the remaining risk factors. The weight function was chosen to mitigate this issue, by limiting the impact of splits deeper in the trees.

Second, identification of conditional risk differences depends on the conditional exchangeability assumption, wherein we assume all variables confounding the effect of SARS-CoV-2 infection on post-acute sick leave are measured and adjusted for by the causal forest. As we cannot rule out the possibility of unmeasured confounders, our results may be attenuated by confounding bias from such variables. Similarly, undetected effect heterogeneity may exist due to unmeasured effect modifiers. This limitation is inherent to causal inference models and has been previously reported in other studies.[10,11]

Supplementary table 1: Characteristics of 88,818 study participants who obtained a positive or negative RT-PCR test for SARS-CoV-2.

Characteristics	Tested negative	Tested positive
	(n=51,336)	(n=37,482)
**Sex**		
Female	34,085 (66.4%)	23,002 (61.4%)
Male	17,251 (33.6%)	14,480 (38.6%)
**Age**		
Mean (SE)	45.8 (13.7)	43.6 (13.9)
Median [Min, Max]	49.0 [15.0, 65.0]	46.0 [15.0, 65.0]
**Charlson Comorbidity Index scores**		
0	45,984 (89.6%)	33,988 (90.7%)
1	2,854 (5.6%)	1,967 (5.2%)
2	1,926 (3.8%)	1,144 (3.1%)
3 or more	572 (1.1%)	383 (1.0%)
**Highest completed education**		
Primary/elementary school	4,267 (8.3%)	2,991 (8.0%)
secondary education or vocational education	5,127 (10.0%)	4,711 (12.6%)
vocational training	8,490 (16.5%)	5,755 (15.4%)
Shorter term higher education	5,999 (11.7%)	4,067 (10.9%)
Medium term higher education	17,237 (33.6%)	12,147 (32.4%)
Long term higher education	8,938 (17.4%)	6,799 (18.1%)
Do not know/None of the above/Do not wish to answer	1,278 (2.5%)	1,011 (2.7%)
**Vaccination status**		
Not vaccinated	51,065 (99.5%)	37,366 (99.7%)
Not fully vaccinated	270 (0.5%)	116 (0.3%)
Primary course	1 (0.0%)	0 (0%)
**Pre-existing conditions (before test)**		
**High BMI**		
No	38,696 (75.4%)	28,599 (76.3%)
Unknown	4,143 (8.1%)	2,680 (7.2%)
Yes	8,497 (16.6%)	6,203 (16.5%)
**Fibromyalgia**		
No	50,862 (99.1%)	37,153 (99.1%)
Yes	474 (0.9%)	329 (0.9%)
**Chronic fatigue syndrome**		
No	50,473 (98.3%)	37,000 (98.7%)
Yes	863 (1.7%)	482 (1.3%)

**Anxiety**		
No	46,905 (91.4%)	34,488 (92.0%)
Yes	4,431 (8.6%)	2,994 (8.0%)
**Depression**		
No	44,870 (87.4%)	33,233 (88.7%)
Yes	6,466 (12.6%)	4,249 (11.3%)
**PTSD**		
No	51,299 (98.0%)	36,762 (98.1%)
Yes	1,037 (2.0%)	720 (1.9%)
**Chronic asthma**		
No	47,840 (93.2%)	34,643 (92.4%)
Yes	3,496 (6.8%)	2,839 (7.6%)
**Diabetes**		
No	49,583 (96.6%)	36,255 (96.7%)
Yes	1,753 (3.4%)	1,227 (3.3%)
**High blood pressure**		
No	45,412 (88.5%)	33,589 (89.6%)
Yes	5,924 (11.5%)	3,893 (10.4%)
**COPD or other lung disease**		
No	50,565 (98.5%)	37,056 (98.9%)
Yes	771 (1.5%)	426 (1.1%)
**Chronic or frequent headaches or migraines**		
No	49,212 (95.9%)	36,013 (96.1%)
Yes	2,124 (4.1%)	1,469 (3.9%)

PTSD: post-traumatic stress disorder, COPD: chronic obstructive pulmonary disease.

Supplementary table 2: Subpopulation counts for combinations of age, high BMI, and depression

	no depression, not high BMI	no depression, high BMI	depression, not high BMI	depression, high BMI
**15-25 yrs**	8,819	513	925	143
**26-35 yrs**	8,860	1,448	1,367	372
**36-45 yrs**	9,864	2,191	1,473	537
**46-55 yrs**	16,103	4,117	1,947	740
**56-65 yrs**	16,284	3,985	1,653	654
**<50 yrs**	33,323	5,525	4,483	1,310
**≥50 yrs**	26,607	6,729	2,882	1,136

The number of persons from subgroups of the study populations defined by combinations of the persons age, BMI, and response regarding existing depression.

Supplementary table 3: Subpopulation counts for combinations of sex, depression, and high BMI

	no depression, not high BMI	depression, not high BMI	no depression, high BMI	depression, high BMI
**female**	37,070	5,394	7,146	1,850
**male**	22,860	1,971	5,108	596

The number of persons from subgroups of the study populations defined by combinations of the persons sex, response regarding existing depression, and BMI.

Supplementary table 4: Covariate balance between study participants with and without a positive RT-PCR test

Characteristics	SMD	Variance ratio	eCDF mean difference	eCDF max difference
**Before/after adjustment**				
**Age**	0.113/0.004	0.97/1.00	0.045/0.001	0.094/0.032
**Sex**	0.074/0.001	·	·	·
**Highest completed education**				
Primary/elementary school	0.009/0.001	·	·	·
secondary education or vocational education	0.058/0.003	·	·	·
Vocational training	0.023/0.003	·	·	·
Shorter term higher education	0.019/0.001	·	·	·
Medium term higher education	0.018/0.000	·	·	·
Long term higher education	0.014/0.001	·	·	·
Do not know	0.009/0.005	·	·	·
**Charlson Comorbidity Index scores**	0.027/0.000	·	·	·
**High BMI**				
Yes	0.000/0.002	·	·	·
No	0.015/0.002	·	·	·
Unknown	0.025/0.001	·	·	·
**Fibromyalgia**	0.003/0.005	·	·	·
**Chronic fatigue syndrome**	0.023/0.011	·	·	·
**Anxiety**	0.016/0.006	·	·	·
**Depression**	0.027/0.006	·	·	·
**PTSD**	0.005/0.002	·	·	·
**Chronic asthma**	0.021/0.003	·	·	·
**Diabetes**	0.006/0.004	·	·	·
**High blood pressure**	0.026/0.004	·	·	·
**COPD or other lung disease**	0.023/0.007	·	·	·
**Chronic headaches or migraines**	0.008/0.001	·	·	·

Covariate balance between study participants with a positive RT-PCR test and those without a positive RT-PCR test before and after inverse propensity weighting. The variance ratio and eCDF statistics are only reported for the continuous age covariate.

Supplementary figure 1: Causal tree example

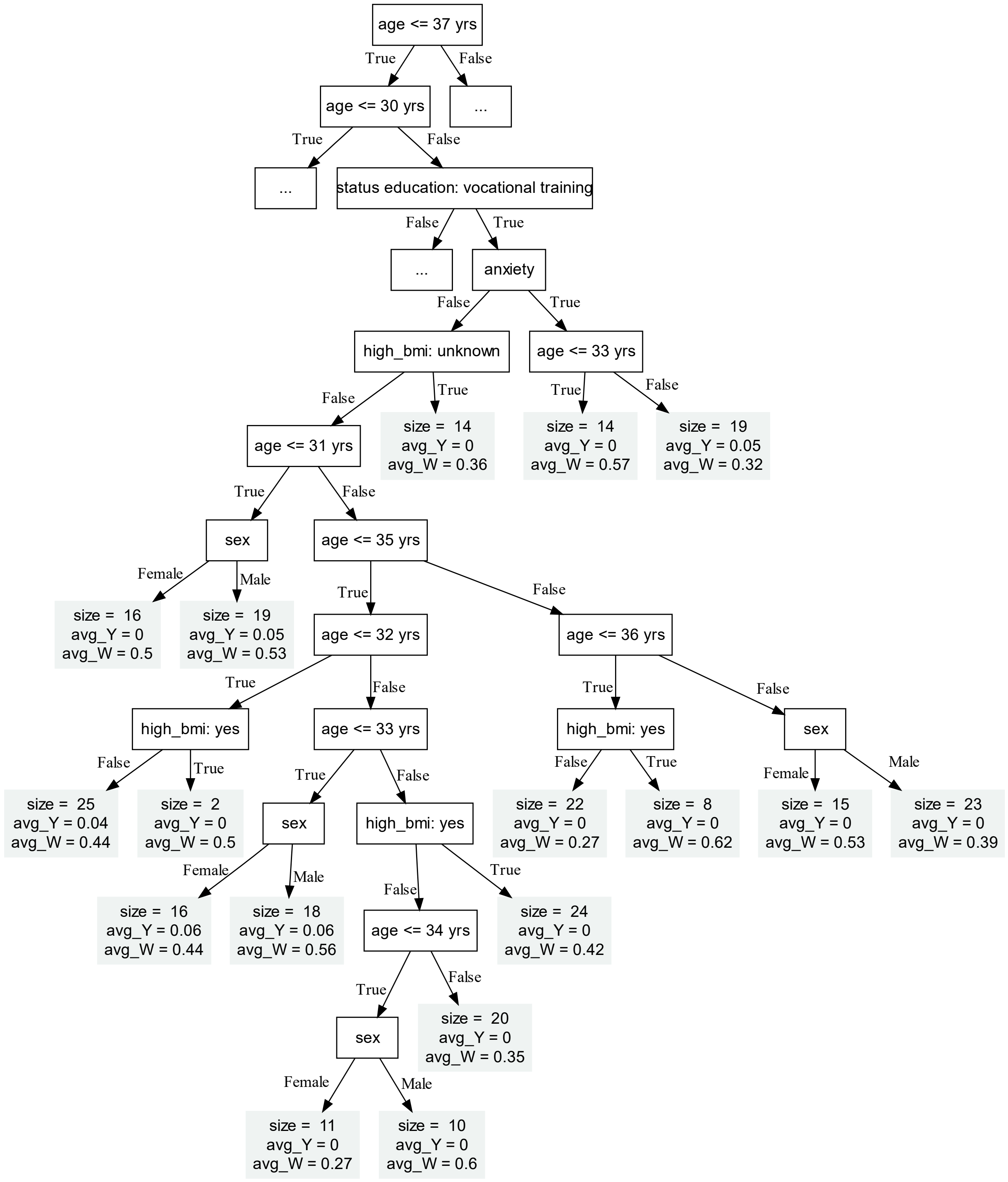

Part of a tree grown for the causal forest model. Splits have been chosen to maximise heterogeneity in the causal effect within the child nodes.

Supplementary figure 2: Causal forest variable importance measure

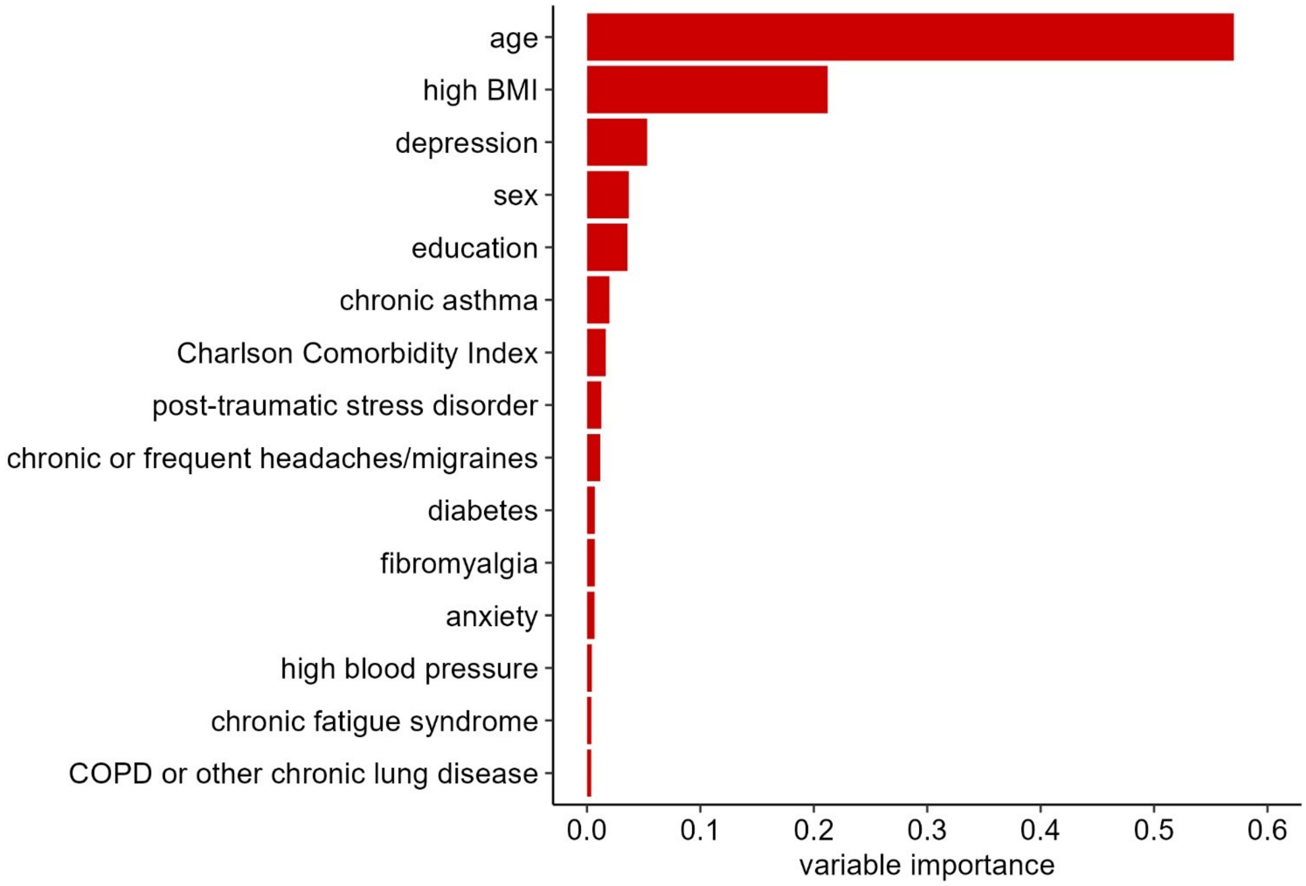

Variable importance measure for the causal forest model. The measure was calculated using a weighted sum of how many times each variable was split at each depth of the tree. The weight function used was the reciprocal squared of the tree depth.

Supplementary figure 3: Estimated risk differences for combinations of sex, high BMI, and age

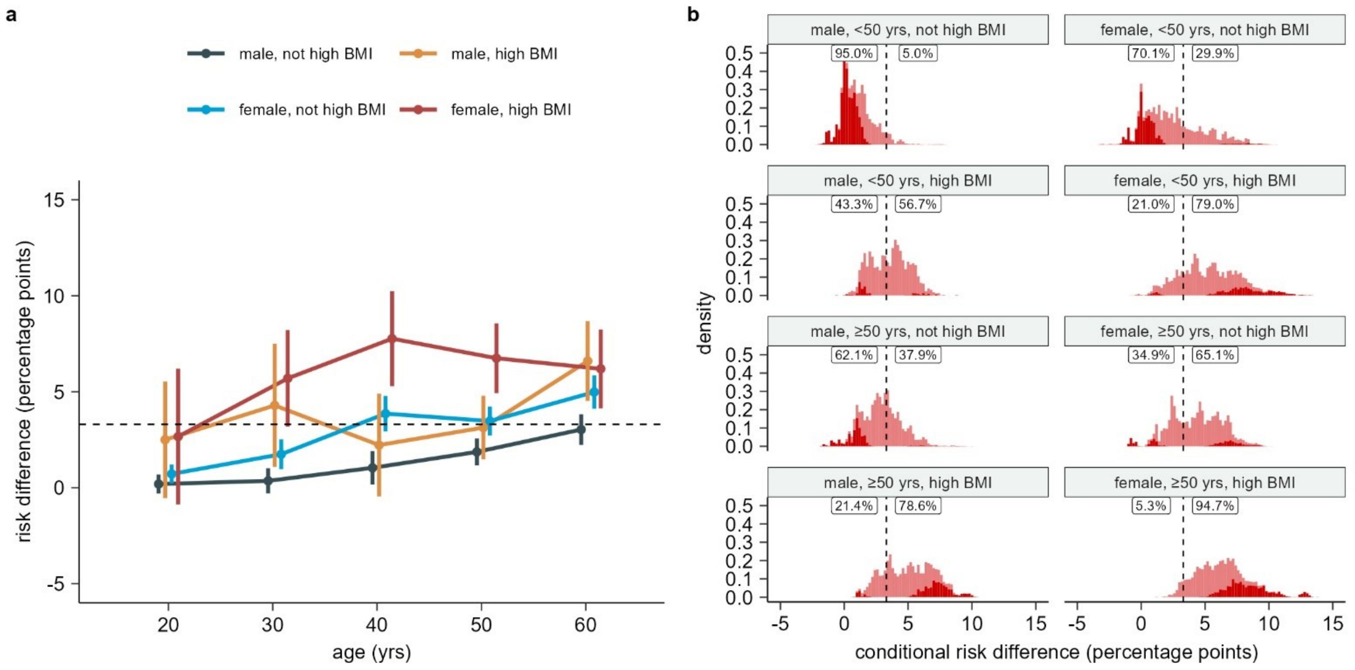

c

	male, not high BMI	male, high BMI	female, not high BMI	female, high BMI
**15-25 yrs**	3310	211	6434	445
**26-35 yrs**	3589	588	6638	1232
**36-45 yrs**	4029	949	7308	1779
**46-55 yrs**	6780	1885	11270	2972
**56-65 yrs**	7123	2071	10814	2568
**<50 yrs**	13316	2335	24490	4500
**≥50 yrs**	11515	3369	17974	4496

a Risk differences (RDs) and two-sided 95% confidence intervals (CI) for full-time sick leave taken four weeks to nine months after the test date between SARS-CoV-2 test-positives and test-negatives for subgroups of a population of n=88,818 individuals. Subgroups are defined by combinations of sex, high BMI, and age risk factors. Subgroups with unknown BMI (n=6,823) are not displayed.

b Distribution of conditional risk differences (CRDs), estimated using out-of-bag prediction from the causal forest model, within subgroups of a population of n=88,818 individuals. Subgroups are defined by combinations of sex, high BMI, and age risk factors. Subgroups with unknown BMI (n=6,823) are not displayed. The dashed line shows the average RD in the full population. The fraction of CRDs below and above this RD is printed for each combination of sex, high BMI, and age. Dark red indicates estimated CRDs significantly different from the average RD in the full population at a 5% significance level using a z-test with two-sided alternative.

c Number of individuals in each of the subgroups appearing in panel **a** and **b**.

Supplementary figure 4: Estimated risk differences for combinations of sex, age, and depression

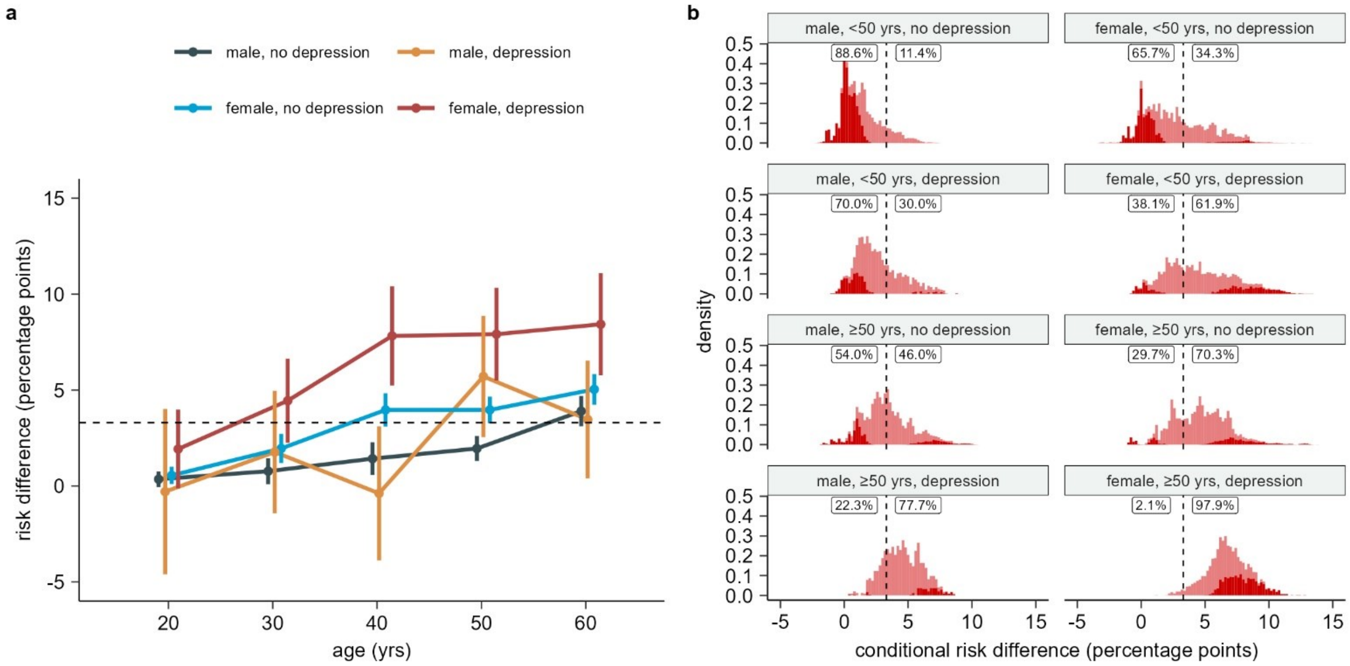

c

	male, no depression	male, depression	female, no depression	female, depression
**15-25 yrs**	3481	247	6790	953
**26-35 yrs**	3888	454	7139	1456
**36-45 yrs**	4656	504	8312	1679
**46-55 yrs**	8202	754	13659	2159
**56-65 yrs**	8819	726	13157	1783
**<50 yrs**	14837	1463	27183	4896
**≥50 yrs**	14209	1222	21874	3134

a Risk differences (RDs) and two-sided 95% confidence intervals (CI) for full-time sick leave taken four weeks to nine months after the test date between SARS-CoV-2 test-positives and test-negatives for subgroups of a population of n=88,818 individuals. Subgroups are defined by combinations of sex, age, and depression risk factors.

b Distribution of conditional risk differences (CRDs), estimated using out-of-bag prediction from the causal forest model, within subgroups of a population of n=88,818 individuals. Subgroups are defined by combinations of sex, age, and depression risk factors. The dashed line shows the average RD in the full population. The fraction of CRDs below and above this RD is printed for each combination of sex, age, and depression. Dark red indicates estimated CRDs significantly different from the average RD in the full population at a 5% significance level using a z-test with two-sided alternative.

c Number of individuals in each of the subgroups appearing in panel **a** and **b**.

Supplementary figure 5: Estimated risk differences for combinations of sex, age, and health conditions.

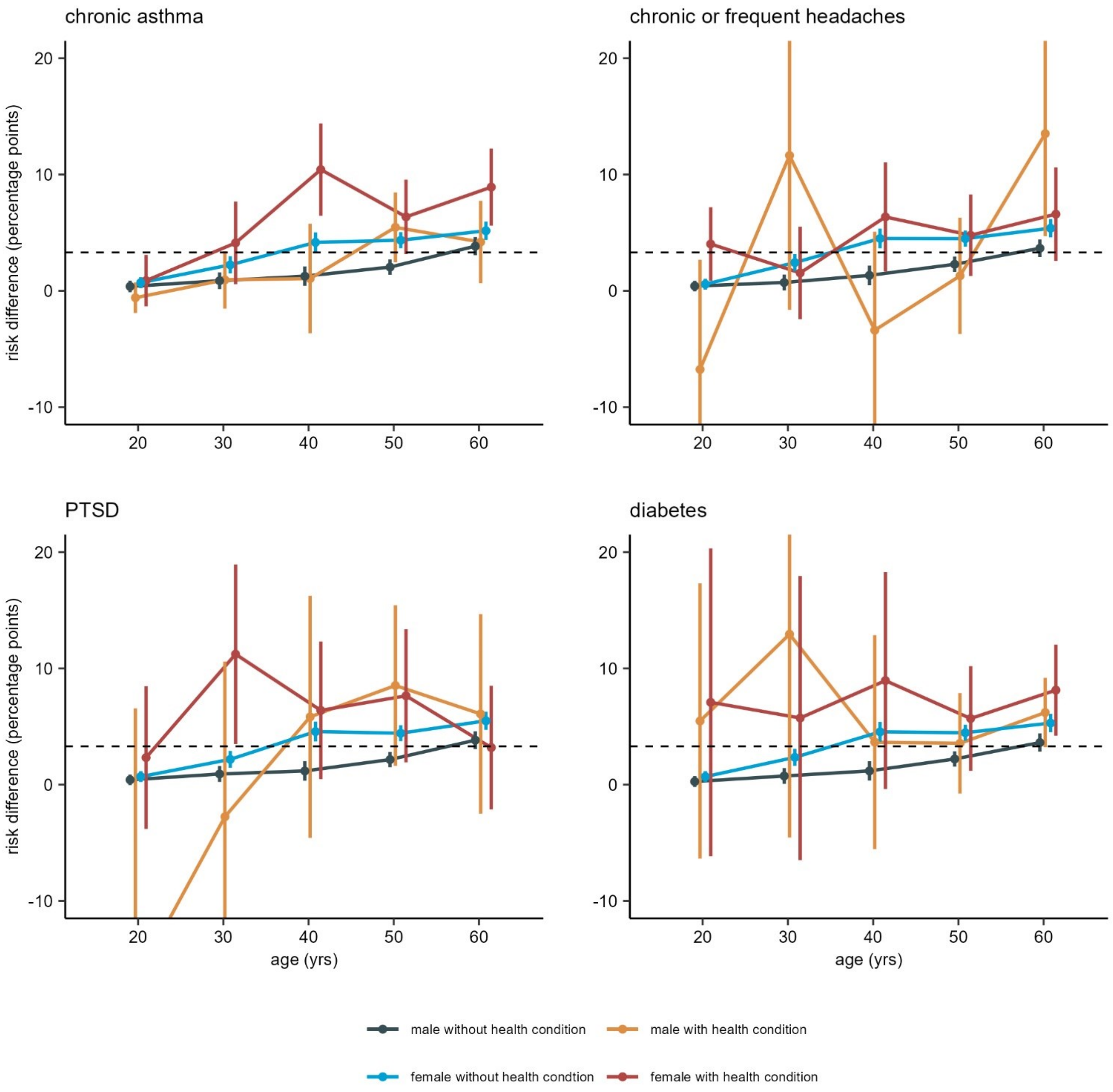

Risk differences (RDs) and two-sided 95% confidence intervals (CI) for full-time sick leave taken four weeks to nine months after the test date between SARS-CoV-2 test-positives and test-negatives for subgroups of a population of n=88,818 individuals. Subgroups are defined by combinations of sex, age, and four different health conditions. Age is grouped in 10-year age groups (15-25 years, 26-35 years, 36-45 years, 46-55 years, 56-65 years).

Supplementary figure 6: Distribution of estimated propensity scores

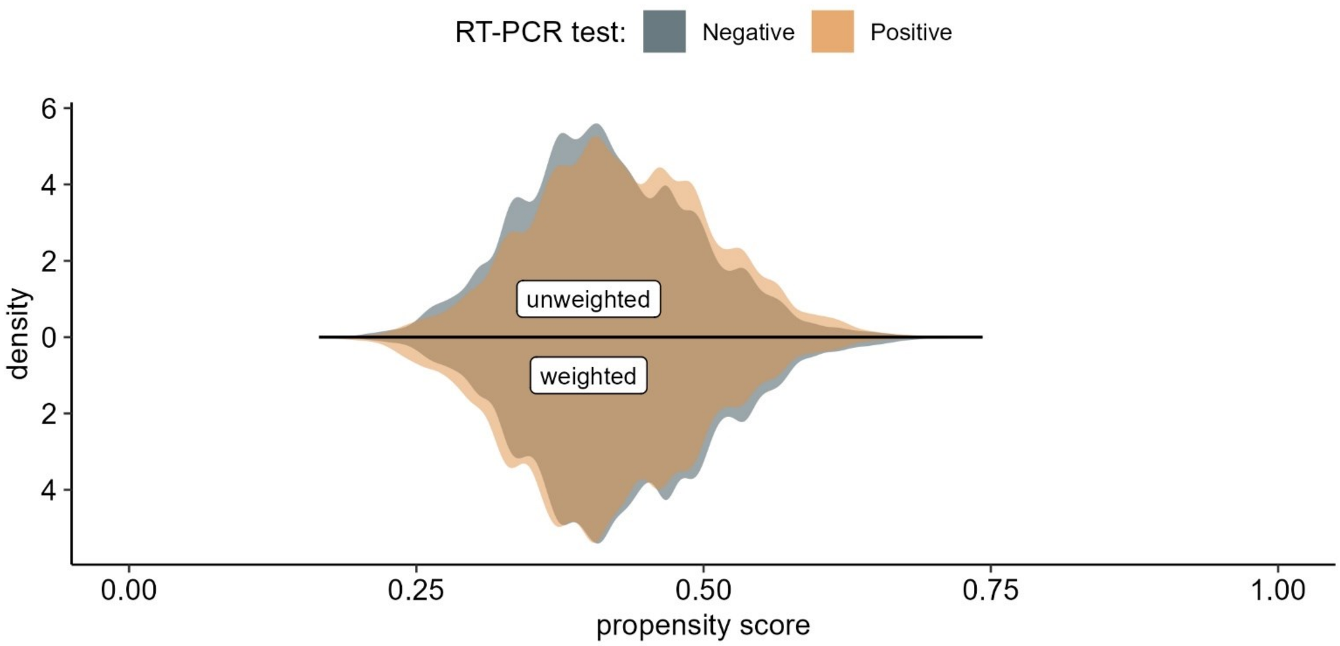

The distribution of propensity scores predicted using a regression forest. The figure shows the distributions within each test group before adjusting with IPW (unweighted) and the distributions after adjusting (weighted). The figure shows good overlap between the test negatives and -positives, and there is no evidence of positivity violations.

Supplementary figure 7: Covariate balance – Love plot

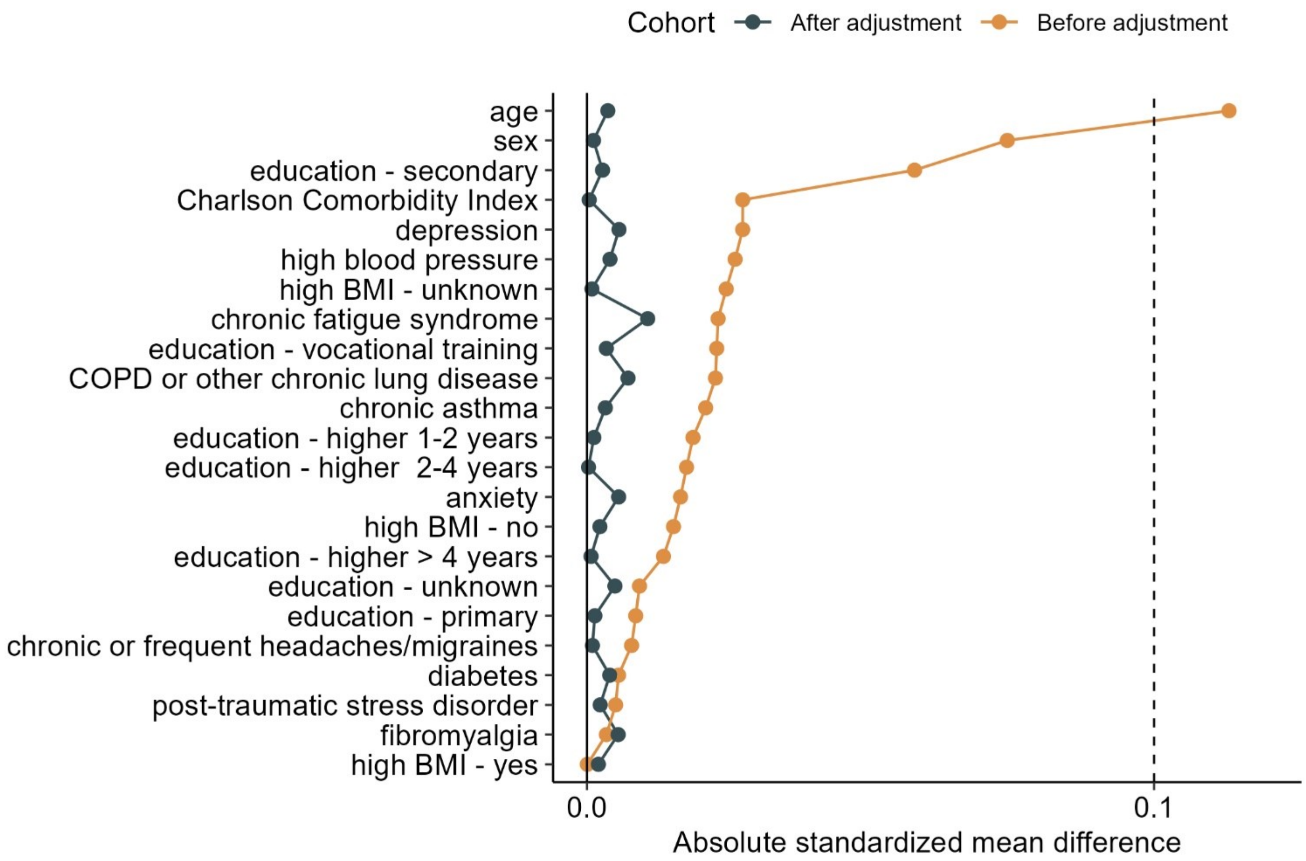

Covariate balance between individuals with a positive RT-PCR test and individuals without a positive RT-PCR test before and after inverse propensity weighting assessed with the absolute standardised mean difference (ASMD). Covariates with ASMD below 0.1 are considered well balanced.

Supplementary figure 8: Covariate balance – eCDF plots

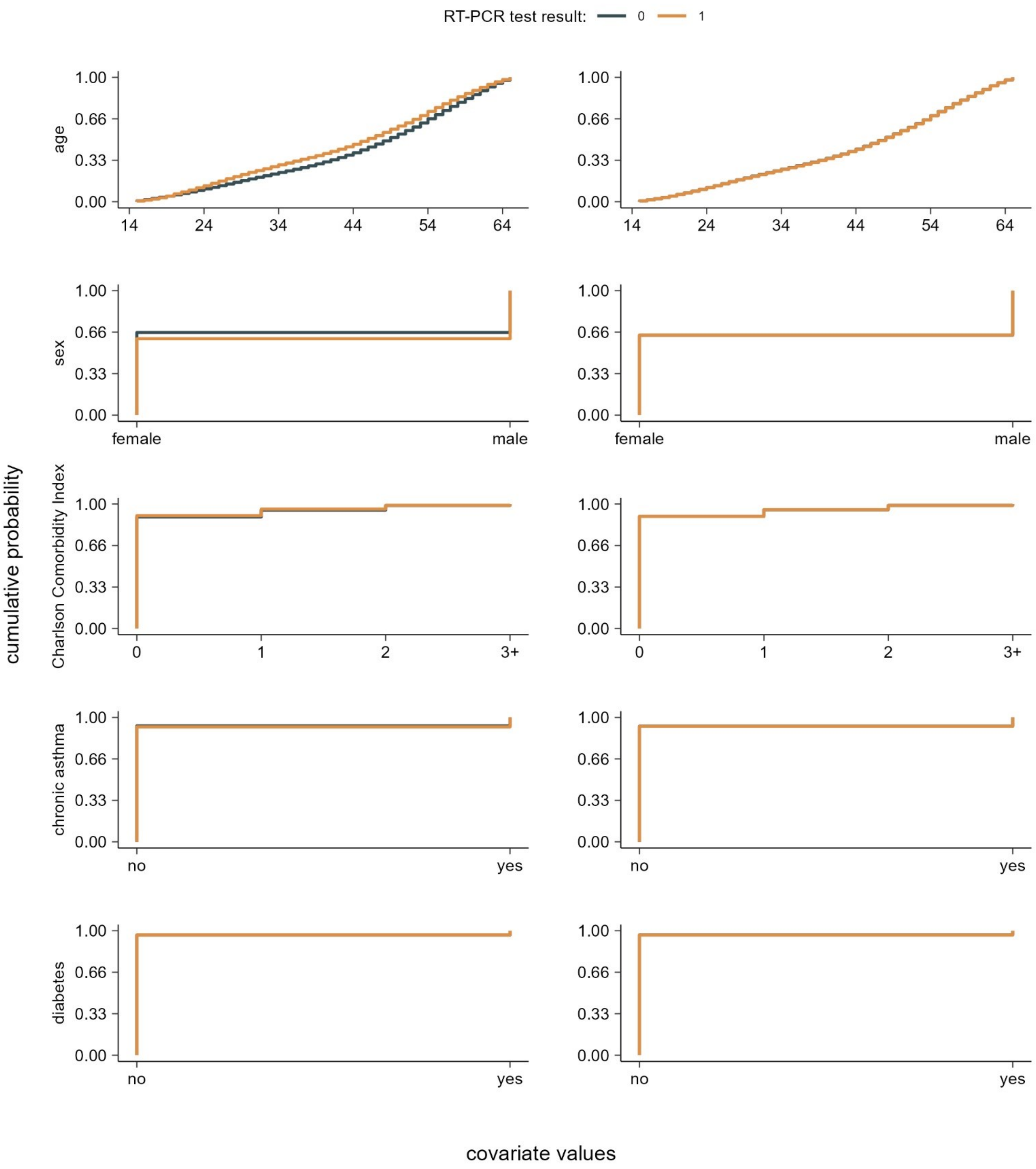

eCDF plots showing balance before (left) and after (right) adjusting with IPW for age, sex, Charlson comorbidity index, chronic asthma, and diabetes.

Supplementary figure 9: Covariate balance – eCDF plots

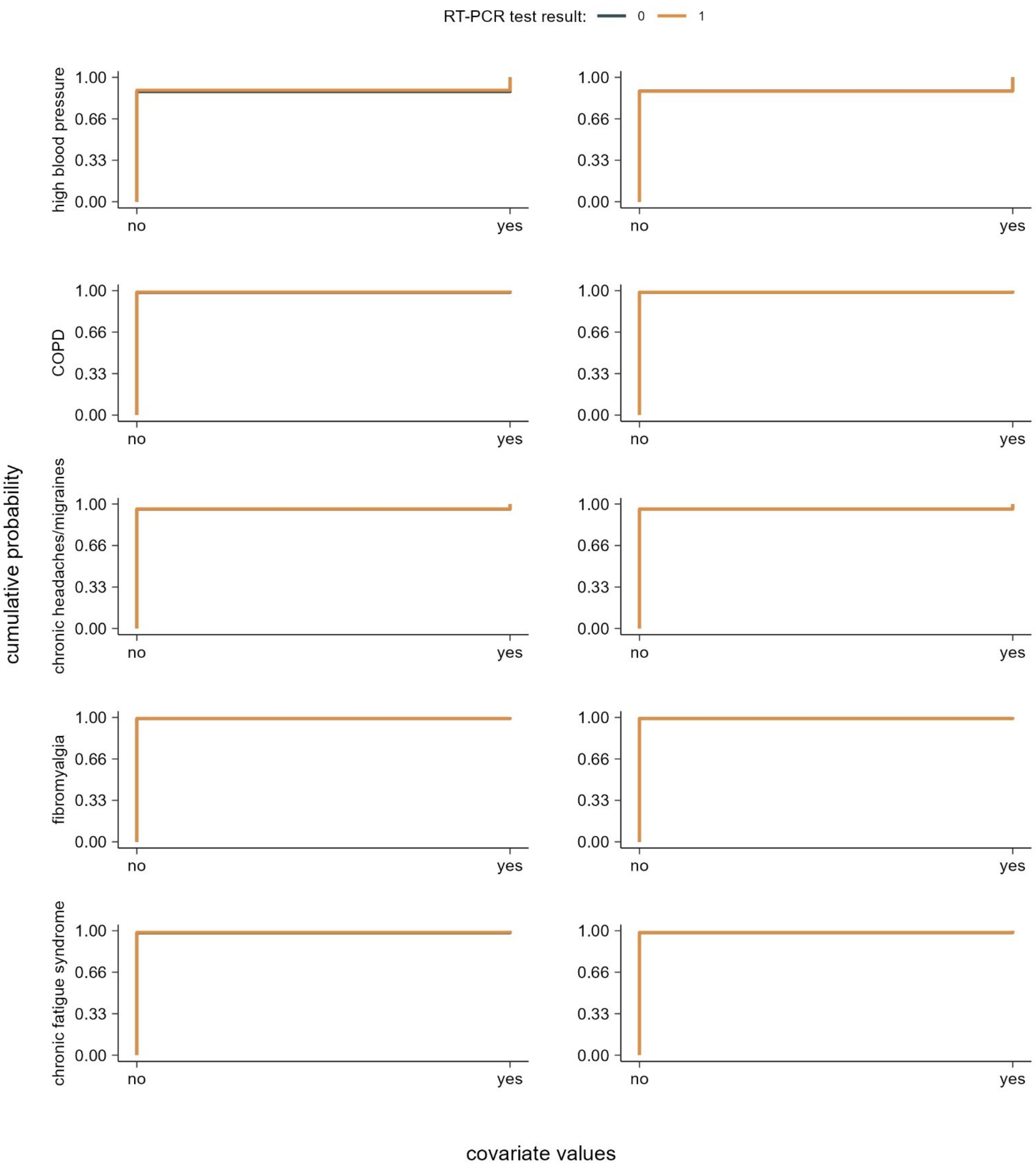

eCDF plots showing balance before (left) and after (right) adjusting with IPW for high blood pressure, COPD or other chronic lung disease, chronic or frequent headaches/migraines, fibromyalgia, and chronic fatigue syndrome.

Supplementary figure 10: Covariate balance – eCDF plots

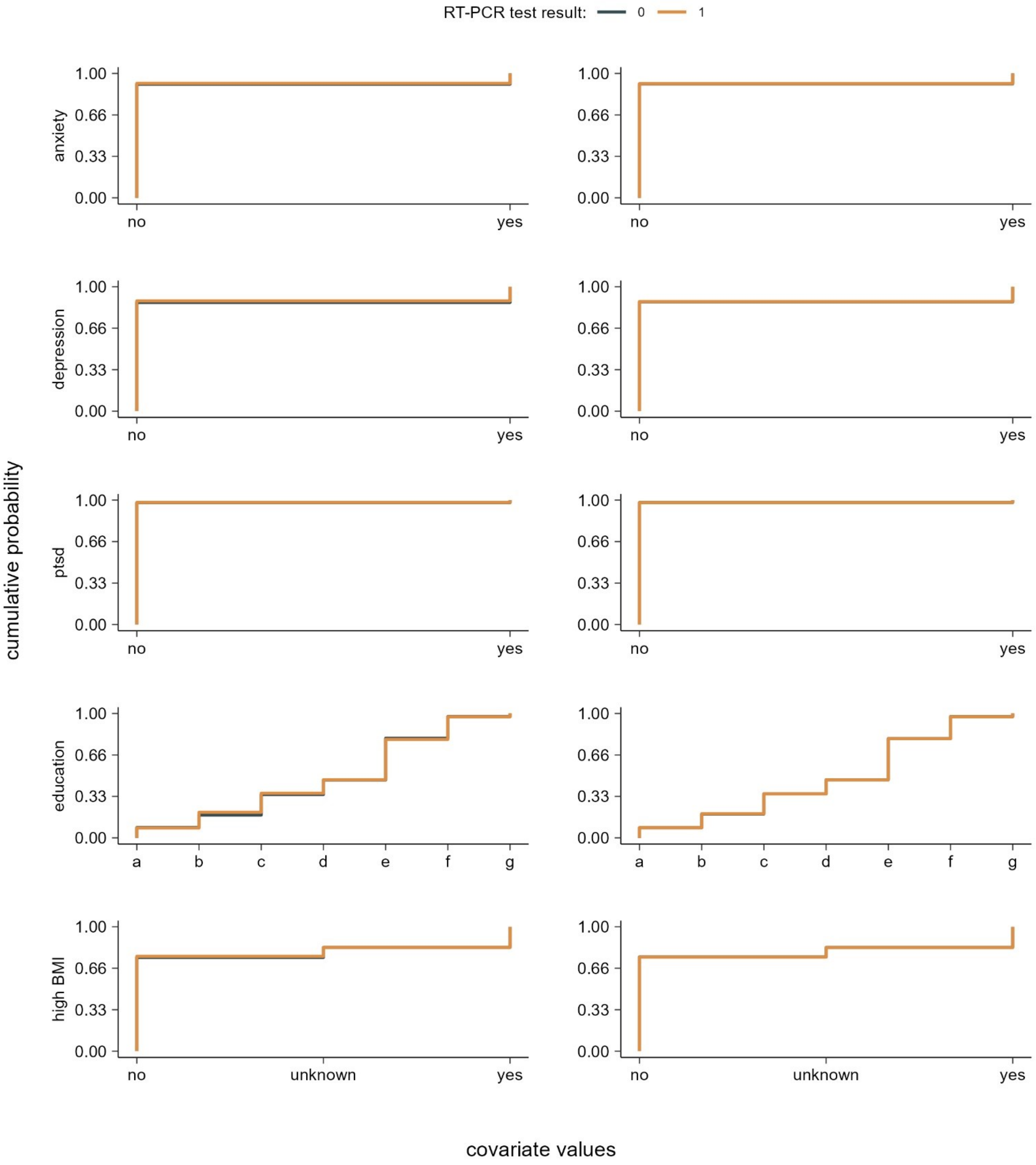

eCDF plots showing balance before (left) and after (right) adjusting with IPW for anxiety, depression, ptsd, education, and high BMI. Education has the following levels: a=primary, b=secondary, c=vocational training, d=higher 1-2 years, e=higher 3-4 years, f=higher ≥5 years, g=unknown.

Supplementary figure 11: RT-PCR test sensitivity analysis - variable importance

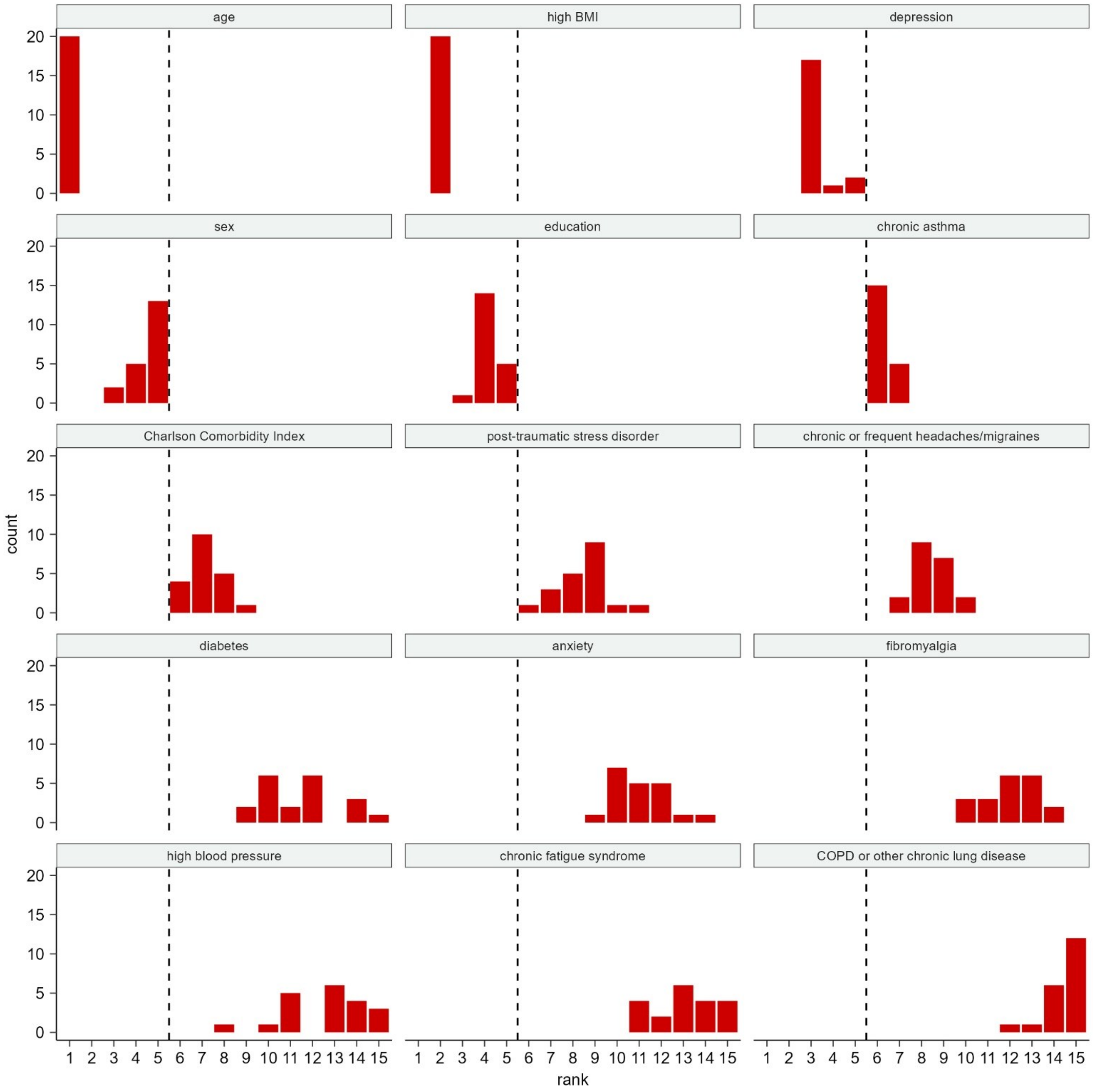

Distribution of variable importance ranking for each risk factor across n = 20 modified exposures.

Supplementary figure 12: Hyperparameter sensitivity analysis - variable importance

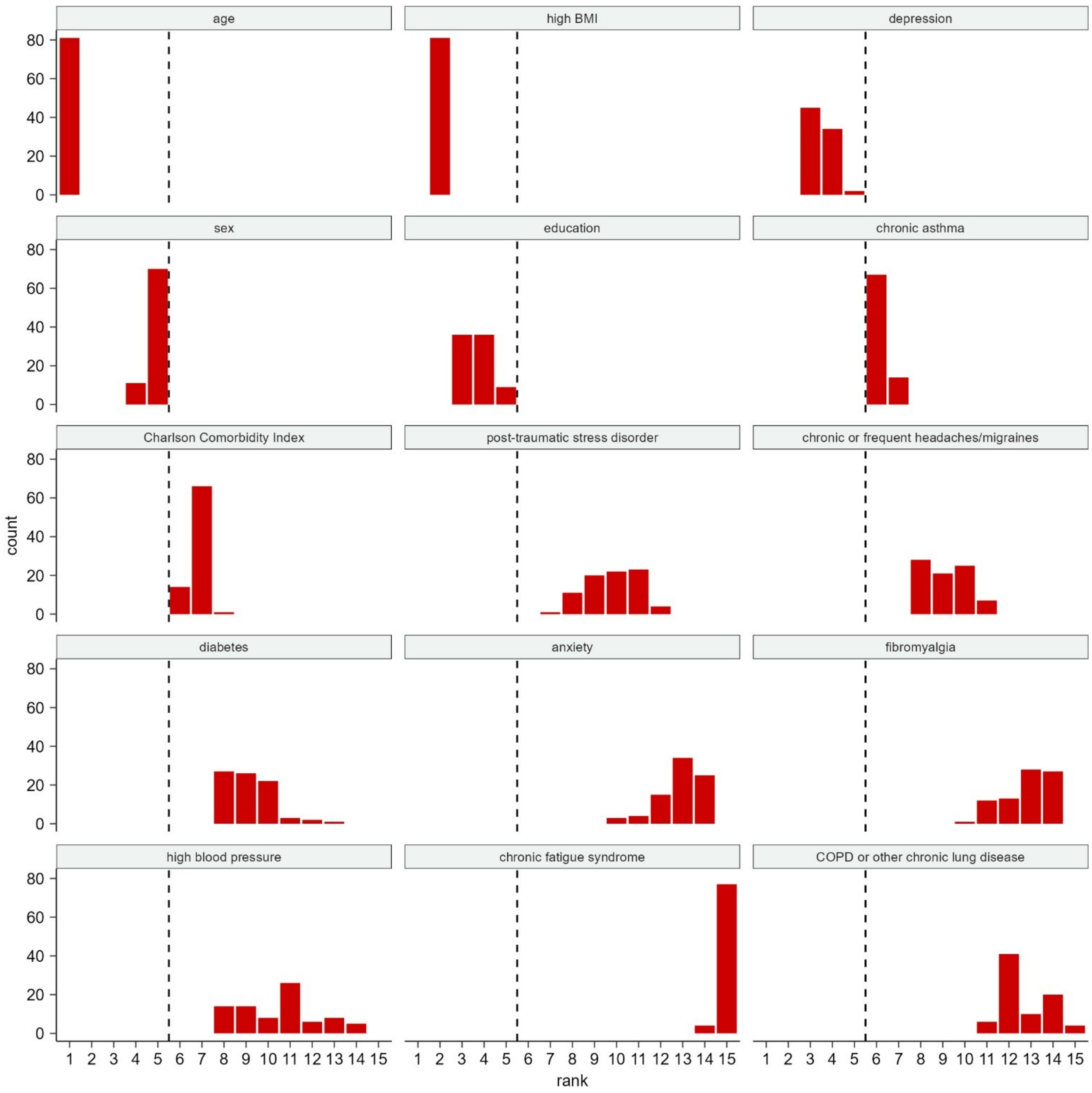

Distribution of variable importance ranking for each risk factor across n = 81 combinations of the hyperparameters *sample fraction, mtry, minimum node size, and honesty fraction.*

Supplementary References

1. Nie, X. & Wager, S. Quasi-oracle estimation of heterogeneous treatment effects. *Biometrika* **108**, 299–319 (2021).

2. Breiman, L. Random Forests. *Mach. Learn.* **45**, 5–32 (2001).

3. Athey, S., Tibshirani, J. & Wager, S. Generalized random forests. *Ann. Stat.* **47**, 1179–1203 (2019).

4. Yadlowsky, S., Fleming, S., Shah, N., Brunskill, E. & Wager, S. Evaluating Treatment Prioritization Rules via Rank-Weighted Average Treatment Effects. http://arxiv.org/abs/2111.07966 (2021) (accessed Oct 9, 2022).

5. Binny, R. N. *et al.* Sensitivity of Reverse Transcription Polymerase Chain Reaction Tests for Severe Acute Respiratory Syndrome Coronavirus 2 Through Time. *J. Infect. Dis.* **227**, 9 (2023).

6. Skittrall, J. P. *et al.* Specificity and positive predictive value of SARS-CoV-2 nucleic acid amplification testing in a low-prevalence setting. *Clin. Microbiol. Infect.* **27**, 469.e9 (2021).

7. Tsampasian, V. *et al.* Risk Factors Associated With Post-COVID-19 Condition: A Systematic Review and Meta-analysis. *JAMA Intern. Med.* (2023) doi:10.1001/jamainternmed.2023.0750.

8. Jacob, L. *et al.* Prevalence of, and factors associated with, long-term COVID-19 sick leave in working-age patients followed in general practices in Germany. *Int. J. Infect. Dis.* **109**, 203 (2021).

9. O'Regan, E. *et al.* A hybrid register and questionnaire study of Covid-19 and post-acute sick leave in Denmark. *Nat. Commun. 2023 141* **14**, 1–8 (2023).

10. Inoue, K., Seeman, T. E., Horwich, T., Budoff, M. J. & Watson, K. E. Heterogeneity in the Association Between the Presence of Coronary Artery Calcium and Cardiovascular Events: A Machine-Learning Approach in the MESA Study. *Circulation* **147**, 132–141 (2023).

11. Inoue, K., Athey, S. & Tsugawa, Y. Machine-learning-based high-benefit approach versus conventional high-risk approach in blood pressure management. *Int. J. Epidemiol.* (2023) doi:10.1093/ije/dyad037.
