# Supplementary material for: Machine learning identifies risk factors associated with long-term sick leave following COVID-19 in Danish population
Source: Commun Med (Lond). 2023 Dec 20;3:188. doi: 10.1038/s43856-023-00423-5 (PMC10733276; doi:10.1038/s43856-023-00423-5)
Supplement: Supplementary file 2 — Reporting Summary [file 43856_2023_423_MOESM2_ESM.pdf]

nature portfolio

Corresponding author(s): Kim Daniel Jakobsen

Last updated by author(s): Aug 28, 2023

Reporting Summary

Nature Portfolio wishes to improve the reproducibility of the work that we publish. This form provides structure for consistency and transparency in reporting. For further information on Nature Portfolio policies, see our Editorial Policies and the Editorial Policy Checklist.

Statistics

For all statistical analyses, confirm that the following items are present in the figure legend, table legend, main text, or Methods section.

n/a	Confirmed	
☐	☒	The exact sample size (*n*) for each experimental group/condition, given as a discrete number and unit of measurement
☐	☒	A statement on whether measurements were taken from distinct samples or whether the same sample was measured repeatedly
☐	☒	The statistical test(s) used AND whether they are one- or two-sided<br>*Only common tests should be described solely by name; describe more complex techniques in the Methods section.*
☐	☒	A description of all covariates tested
☐	☒	A description of any assumptions or corrections, such as tests of normality and adjustment for multiple comparisons
☐	☒	A full description of the statistical parameters including central tendency (e.g. means) or other basic estimates (e.g. regression coefficient) AND variation (e.g. standard deviation) or associated estimates of uncertainty (e.g. confidence intervals)
☐	☒	For null hypothesis testing, the test statistic (e.g. *F*, *t*, *r*) with confidence intervals, effect sizes, degrees of freedom and *P* value noted<br>*Give P values as exact values whenever suitable.*
☒	☐	For Bayesian analysis, information on the choice of priors and Markov chain Monte Carlo settings
☒	☐	For hierarchical and complex designs, identification of the appropriate level for tests and full reporting of outcomes
☒	☐	Estimates of effect sizes (e.g. Cohen's *d*, Pearson's *r*), indicating how they were calculated

Our web collection on statistics for biologists contains articles on many of the points above.

Software and code

Policy information about availability of computer code

Data collection	The web-based questionnaires used for data collection were created in SurveyXact (www.surveyxact.dk). The SurveyXact system is used directly online and no version number exists.
Data analysis	The statistical analyses were carried out in R version 4.2.2. The R-package grf version 2.2.1 was used for modelling and ggplot2 version 3.4.2 was used for visualisations.

For manuscripts utilizing custom algorithms or software that are central to the research but not yet described in published literature, software must be made available to editors and reviewers. We strongly encourage code deposition in a community repository (e.g. GitHub). See the Nature Portfolio guidelines for submitting code & software for further information.

Data

Policy information about availability of data

All manuscripts must include a data availability statement. This statement should provide the following information, where applicable:

- Accession codes, unique identifiers, or web links for publicly available datasets
- A description of any restrictions on data availability
- For clinical datasets or third party data, please ensure that the statement adheres to our policy

The datasets used in this study comprise sensitive, individual-level information from completed questionnaires and national register data. According to the Danish data protection legislation, the authors are not permitted to share these sensitive data directly upon request. However, the data are available for research purposes

upon request to the Danish Health Authority (register data, email: kontakt@sundhedsdata.dk) and Statens Serum Institut (questionnaire data, email: aii@ssi.dk), as well as within the framework of the Danish data protection legislation and any required permission from authorities. Data request processing can take an expected three to six months.

Human research participants

Policy information about studies involving human research participants and Sex and Gender in Research.

Reporting on sex and gender	Sex and gender were not considered in the design of the study. Sex was used in data analysis as a potential confounder and effect modifier of the effect of COVID-19 infection on substantial post-acute full-time sick leave. Gender was not used in the data analysis since it could not be asertained from the EFTER-COVID questionnaire. The sex was identified from the unique identifier (CPR-number) in the Danish Civil Registration System assigned to all Danish residents.
Population characteristics	The study cohort consisted of 88,818 individuals, of which 37,482 had had a confirmed SARS-CoV-2 infection. The mean age was 45 years with standard error 14 years and 64.3% were female. 62.1% had some form of higher education, while 16.0% had vocational training. The most prevalent existing health conditions before test were high BMI (16.6%), depression (12.1%), high blood pressure (11.1%), and anxiety (8.4%).
Recruitment	Participants in the EFTER-COVID study received a positive RT-PCR test result between November 4, 2020 and February 1, 2021 or were randomly selected among test-negatives using incidence density sampling on the test date with a ratio of 2:3 between test-positive and -negative persons. Participants were recruited if they were alive and living in Denmark 9 months after their test date, were registered with the national mail system, e-Boks, and did not have a positive test result less than 9 months after their test date.
Ethics oversight	This study was performed as a surveillance study as part of the governmental institution Statens Serum Institut's (SSI) advisory tasks for the Danish Ministry of Health. SSI's purpose is to monitor and fight the spread of disease in accordance with section 222 of the Danish Health Act. According to Danish law, national surveillance activities carried out by SSI do not require approval from an ethics committee.

Note that full information on the approval of the study protocol must also be provided in the manuscript.

Field-specific reporting

Please select the one below that is the best fit for your research. If you are not sure, read the appropriate sections before making your selection.

☒ Life sciences ☐ Behavioural & social sciences ☐ Ecological, evolutionary & environmental sciences

For a reference copy of the document with all sections, see nature.com/documents/nr-reporting-summary-flat.pdf

Life sciences study design

All studies must disclose on these points even when the disclosure is negative.

Sample size	The sample size was not pre-determined. The final sample size is based on the number of eligible individuals with a positive RT-PCR tests in the study period, as all these were recruited for the study.
Data exclusions	The exclusion criteria were pre-established. Individuals were excluded if they did not complete the questionnaire, indicated they believed they had been previously infected with SARS-CoV-2 due to receiving a seropositive result for SARS-CoV-2, or were >65 years-old at the time of the test. Exclusion based on previous infection was done to ensure a control group without potential post COVID-19 condition. Exclusion based on age was done to restrict the study to the working age population.
Replication	The present study is a questionnaire study and has not been replicated. Instead, we have compared our results to results from other sources and found them reasonably similar. An English translation of the survey has previously been made available for others to use if they wish to repeat the study.
Randomization	Allocation into the experimental groups was not random. All persons available to invitation who had recieved a positive PCR results within the study period were invited to participate. Controls were randomly selected among persons available to invitation who had recieved a negative PCR result using incidence density sampling on the test date with a ratio of 2:3 between test-positive and -negative persons. The covariates age, sex, Charlson Comorbidity Index, education level, chronic asthma, diabetes, high blood pressure, COPD or other chronic lung disease, chronic or frequent headaches/migraines, fibromyalgia, chronic fatigue syndrome, anxiety, depression, post-traumatic stress disorder, and high BMI were controlled for by allowing the causal forest model used to estimate risk differences to place splits along these variables.
Blinding	Blinding was not relevant to this study, since it used observational data, where exposure groups are not randomly allocated.

Reporting for specific materials, systems and methods

We require information from authors about some types of materials, experimental systems and methods used in many studies. Here, indicate whether each material, system or method listed is relevant to your study. If you are not sure if a list item applies to your research, read the appropriate section before selecting a response.

Materials & experimental systems

n/a	Involved in the study
☒	☐ Antibodies
☒	☐ Eukaryotic cell lines
☒	☐ Palaeontology and archaeology
☒	☐ Animals and other organisms
☒	☐ Clinical data
☒	☐ Dual use research of concern

Methods

n/a	Involved in the study
☒	☐ ChIP-seq
☒	☐ Flow cytometry
☒	☐ MRI-based neuroimaging
